# Factor structure of the depression anxiety stress Scale-21 (DASS-21): Unidimensionality of the Arabic version among Egyptian drug users

**DOI:** 10.1186/s13011-019-0226-1

**Published:** 2019-09-18

**Authors:** Amira Mohammed Ali, Joseph Green

**Affiliations:** 1 Department of Psychiatric Nursing and Mental Health, Faculty of Nursing, Alexandria University, Alexandria, Egypt; 2 Department of Mental Disorder Research, National Institute of Neuroscience, National Center of Neurology and Psychiatry, 4-1-1, Ogawahigashi, Kodaira, Tokyo, 187-8502 Japan; 3 The Graduate School of Medicine, The University of Tokyo, Tokyo, Japan

**Keywords:** DASS-21, Factor analysis, Validation, Drug related disorders, Parallel analysis

## Abstract

**Background:**

Emotional distress is common among illicit drug users, and it can negatively affect treatment outcomes and increase the risk of relapse. Nonetheless, instruments that properly measure emotional distress are lacking. Therefore, this study investigated the factor structure of the Arabic Depression Anxiety Stress Scale-21 (DASS-21) in that population.

**Methods:**

The DASS-21 and the Self-stigma of Alcohol Dependence Scale (SSAD) were completed by 149 inpatient Egyptian drug users. The DASS-21 was examined using exploratory factor analysis, partial confirmatory factor analysis, and parallel analysis. For validation testing, correlations between stigma scores and DASS scores were computed.

**Results:**

A one-factor solution provided the best fit to the DASS-21 data. Four items with low loadings were removed. The resulting DASS-17 was also unidimensional, and its reliability was high (0.88). On the validation tests, the DASS scores correlated with the stigma scores as hypothesized.

**Conclusion:**

Subscales of the Arabic version of the DASS-21 do not differentiate between depression and anxiety. A modified 17-item version (the DASS-17) was suitable for measuring overall distress, and the results of convergent validation testing indicated that it was superior to the DASS-21.

## Background

Depressive and anxiety disorders are common worldwide, affecting, respectively, 322 million and 264 million, equivalent to 4.4% and 3.6% of the world’s population [1]. The prevalence of depressive and anxiety symptoms and their concurrence is even higher ranging from 9.3% to 27.2% [2–4]. Low- and lower-middle income countries witness the highest prevalence where poverty and economic pressure prevail [5]. Depression and anxiety are associated with several social and physical problems such as disturbed family relations, high suicidality (more than 800,000 per year), poor academic performance, and use of illicit drugs [1–4, 6, 7].

More than three quarters of drug users have symptoms of depression and anxiety (Moody, Franck, & Bicke, 2016); the severity of symptoms varies between those in long-term residential treatment and those in outpatient treatment [8]. Their psychological distress is associated with low abstinence self-efficacy, craving, treatment failure, relapse, and continued use [9, 10], but the frequency of drug use decreases when negative emotions decline during treatment [11]. Therefore, measuring emotional negativity in people with substance use disorders is essential to evaluate and improve treatment outcomes and possibly prevent relapse [12].

Although a large number of scales that measure anxiety and/or depression exists, these measures fail to differentiate between both constructs [13]. The Depression Anxiety Stress Scale (DASS) is a 42-item tool for assessing the discrete features of depression, anxiety, and stress [14]. The aim of its development was to reduce the measurement overlap between depression and anxiety that threatens the purity of standalone measures of depression or anxiety [15]. Its 3-factor structure ideally matches the tripartite classification of depression and anxiety symptoms: non-specific symptoms of general distress, anhedonia/low positive affect specific to depression, and somatic arousal specific to anxiety [16]. Because the short version (DASS-21) is relatively easy to administer, it has been broadly used for research and clinical purposes in various groups and settings [15, 17, 18]—including people with substance use disorders [19].

Psychometric properties of the full version are well-established [14, 20]. However, for the DASS-21 several studies reported inconsistent findings in different cultural contexts (including English speaking ones where it was originally developed) and via various quality assessment techniques [5, 13, 15, 17, 18, 21–28]. Similarly, examinations of scalability and item functioning indicate that the Arabic version of the DASS-21 contains some problematic items with regard to discrimination, level of difficulty, and invariance across different groups [26, 29]. Meanwhile, the factor analysis of the Arabic DASS-21 has not been examined neither remedial actions were carried out to address erroneous items. Therefore, the current study aims to report the results of factor analysis, reliability testing, and convergent validation testing of the Arabic DASS-21.

Convergent or construct validity examines whether constructs that should be related are related [30]. Deriving from previous reports which indicate that self-stigma—negative self-views: awareness of stereotypes (negative public attitudes toward substance users), personal agreement with public stereotypes, self-occurrence (application of negative public attitudes to self), and shame (loss of self-esteem because of flawed self-evaluations) [31]—lead to unpleasant emotions [32], bivariate correlation between the DASS-21 and Self-Stigma in Alcohol Dependence Scale was used to test convergent validity (described in details latter in the Methods) [30]. Specifically, we hypothesized that scores on the stigma subscales measuring awareness of public stereotypes and agreement to them would be only weakly correlated with the DASS-21 score, because awareness of public stereotypes and agreement to them reflect knowledge and cognitive processes rather than emotions. In contrast, we hypothesized that scores on the stigma subscales measuring stereotype self-occurrence and drug-related shame (which reflect affective aspects of stigma that are more related to psychological symptoms) would positively correlate with scores on the DASS-21.

## Methods

### Participants

This study recruited people who were being treated for substance use disorders and were inpatients at a government psychiatric hospital in Alexandria, Egypt. Eligible patients were included if they could read and write, were free from severe mental disorders (e.g., schizophrenia, suicidal ideation), and gave written informed consent. Of 420 inpatients, 51.2% were eligible to participate, but only 35.5% took part in the study. The sample comprised 149 participants (95.3% men, mean age = 32.5 years, SD = 6.8 years, age range: 19–60 years). Heroin, synthetic drugs, and cannabis were the most commonly used drugs 80.5, 79.2, and 75.2%, respectively (see Table 1 for sociodemographic and clinical characteristics of the participants). The current study is a secondary analysis of a former study on self-stigma of substance use disorders [33]. The board of research ethics of Faculty of Nursing, University of Alexandria, approved the study, and participants provided a written informed consent prior to data collection.

**Table 1:** Sociodemographic and clinical characteristics (*n* = 149)

Characteristic	*n* (%)
Age	
≤ 30 years	72 (48.3)
31–40 years	61 (40.9)
> 40 years	16 (10.7)
Mean (SD) in years	32.5 (6.8)
Gender	
Males	142 (95.3)
Females	7 (4.7)
Marital status	
Single	74 (49.7)
Married	55 (36.9)
Others	20 (13.4)
Education	
High school or less	114 (76.5)
Above high school	35 (23.5)
Employment	
Employed	110 (73.8)
Unemployed	39 (26.2)
Income (*n* = 148)^a^	
Enough	104 (69.8)
Not enough	44 (29.5)
Lifetime substance use^b^	
Cannabis	112 (75.2)
Bango	40 (26.8)
Heroin	120 (80.5)
Synthetic drugs	118 (79.2)
Alcohol	62 (41.6)
Others	31 (20.8)
Chronicity	
≤ 10 years	50 (33.6)
20 years –	75 (50.3)
≥ 30 years	24 (16.1)
Mean (SD) in years	14.4 (7.1)
History of mental illness (*n* = 147)	
Yes	100 (67.1)
No	47 (31.5)
Hospital stay	
≤ 15 days	39 (26.2)
16–30 days	73 (49.0)
> 30 days	37 (24.8)

^a^Income was subjectively assessed by rating it as either enough or not enough. ^b^Participants used several substances concurrently

### Instruments

#### The depression anxiety stress scale–21 (DASS–21)

The DASS-21 has 21 items in 3 subscales of 7 items each. They ask about depressive symptoms (e.g., feeling down-hearted and blue), anxiety symptoms (e.g., feeling close to panic), and general stress symptoms (e.g., having a tendency to over-react to situations). Response options are on a 4-point scale (0 = *did not apply to me at all* and 3 = *applied to me most of the time)*. Higher scores indicate more psychological distress [34].

#### The self-stigma in alcohol dependence scale (SSAD)

The SSAD uses 5-point Likert-type responses. It has four subscales of 16 items each. It measures four aspects of stigma: awareness of public stereotypes, stereotype agreement, stereotype self-occurrence, and drug-related shame [31]. This scale was translated into Arabic and modified by substituting “substance dependence” for “alcohol dependence” since participants used alcohol and other drugs. Estimates of internal consistency for the translated version were adequate (coefficient alpha = 0.81, 0.86, 0.83, and 0.84 for the four subscales). These indices of stigma were used for validation testing. All questionnaires were self-administered.

### Statistical analyses

Data from the DASS-21 were analyzed in 2 stages. First, to check if the data fit the 3-factor structure of the DASS-21 set by Lovibond and Lovibond (1995), items’ loadings of the DASS-21 were examined after exploratory factor analysis (EFA), retaining the number of factors with an eigenvalue greater than one. Then, a set of EFA was conducted using maximum-likelihood extraction and direct oblimin rotation with Kaiser normalization. The number of factors was set to 4, 3, and 2. The χ^2^ and df values were obtained from Bartlett’s test of sphericity and also from the Goodness-of-Fit test, and they were used to compute the normed fit index (NFI), the comparative fit index (CFI), the Tucker-Lewis index (TLI) (all should be > 0.95), and the root mean square error of approximation (RMSEA, which should be < 0.08) [35]. Parallel analysis — principal components analysis with raw data permutation — [36] was performed to determine the number of components to extract. In the second stage, items 2, 6, 14, and 18 were removed because their factor loadings were less than 0.4 [37] and then ran EFA and parallel analysis.

For the full DASS-21 and the modified version (DASS-17), reliability indices were computed: alpha coefficients, corrected item-total correlations, and the values of alpha if an item was deleted. As for validation tests, correlations between the SSAD subscale scores and the DASS-21 and DASS-17 scores were computed to test the hypotheses described above. The data were analyzed with SPSS version 22.

## Results

### Factor analysis

**Fig. 1:**
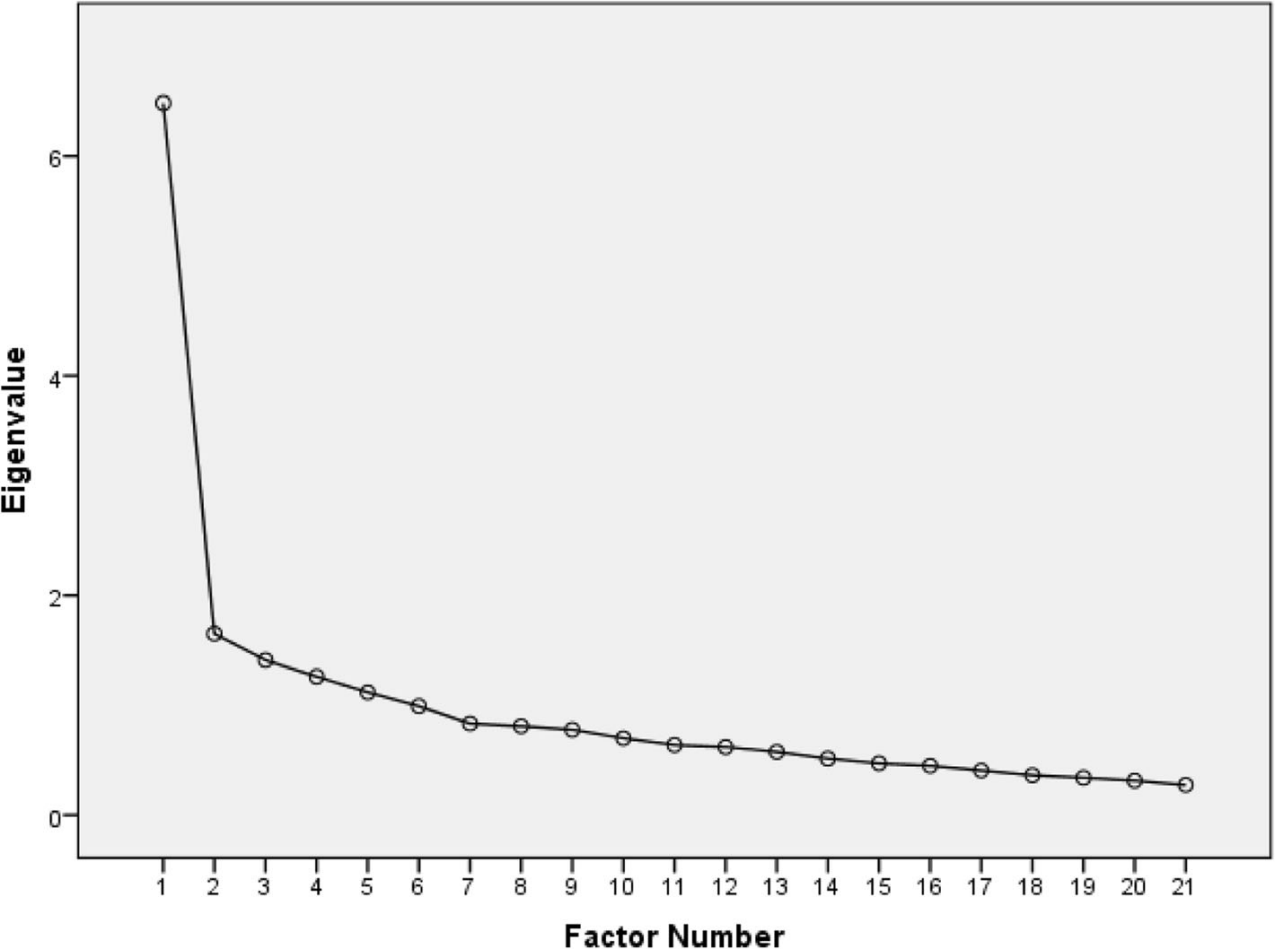
Scree plot of eigenvalues from un-rotated EFA of the DASS-21

In the initial EFA of the DASS-21, 5 factors had eigenvalues greater than 1 (6.48, 1.65, 1.41, 1.26, and 1.12). The scree plot had a rather distinct “elbow” at the second factor (Fig. 1). Table 2 shows loadings for both the 3-factor and 1-factor solutions. In the 3-factor model, almost all items had their highest loadings on the first factor. The only exceptions were items 2 and 3, which cross-loaded on the third and second factors, respectively. In the 1-factor model, similar to the 3-factor model, items 2, 6, 14, and 18 had the lowest loadings (all below 0.4) and the lowest communalities (0.196, 0.133, 0.108, and 0.145). The model fit indices indicated failure of the DASS-21 items to load on 4, 3, or 2 factors—the NFI, in particular, was substantially low: 0.84, 0.80, and 0.75 for 4, 3, and 2 factors, respectively (Table 3).

**Table 2:** Results of exploratory factor analysis of the DASS-21 and the DASS-17 (extraction by maximum likelihood)

Item number	Loadings of 3-factors solution^a^			Loadings of 1-factor solution, DASS-21	Loadings of 1-factor solution, DASS-17
	Factor 1	Factor 2	Factor 3		
1	.535	−.046	−.057	.531	.528
2	.312	−.182	.307	.317	
3	.454	.580	−.207	.439	.447
4	.624	.037	.155	.639	.626
5	.505	.164	.228	.525	.510
6	.351	.090	.245	.363	
7	.452	−.346	.255	.452	.429
8	.461	.078	−.139	.456	.440
9	.501	−.265	.093	.494	.491
10	.606	.260	.057	.614	.617
11	.508	.018	.359	.529	.503
12	.616	−.029	.286	.632	.624
13	.619	.173	−.035	.623	.640
14	.312	−.004	.200	.320	
15	.540	−.153	.087	.539	.540
16	.508	.242	.065	.515	.524
17	.608	.144	−.187	.598	.614
18	.364	−.042	.126	.368	
19	.559	−.156	−.037	.554	.546
20	.656	−.400	−.362	.583	.590
21	.733	.065	−.170	.710	.706

*DASS* Depression Anxiety Stress Scale, ^a^Oblimin rotation with Kaiser normalization. After that oblique rotation, the correlations among the three factors were as follows: r(F1,F2) = −0.31, r(F1,F3) = −0.28, and r(F2,F3) = 0.56

**Table 3:** Partial confirmatory factor analysis for the DASS-21

Factors	Null χ^2^	Null df	Implied χ^2^	Implied df	NFI	CFI	TLI	RMSEA
Two	973.5	210	247.6	169	0.75	0.90	0.87	0.06
Three	973.5	210	199.1	150	0.80	0.94	0.91	0.05
Four	973.5	210	155.2	132	0.84	0.97	0.95	0.03

Null χ^2^ and null df come from Bartlett’s test of sphericity, implied χ^2^ and implied df come from the Goodness-of-Fit test. *NFI* Normed fit index, *CFI* Comparative fit index, *TLI* Tucker-Lewis index, *RMSEA* Root mean square error of approximation

In the parallel analysis (Table 4), only 1 component had an eigenvalue that was above the 95th percentile of eigenvalues of 1000 random datasets of the same dimension. The eigenvalue of that component was 6.48, which is equal to the eigenvalue of the first factor extracted in the initial EFA, which explained 30.9% of the common variance.

**Table 4:** Parallel analysis (principle components analysis with the method of raw data permutation) of the DASS-21 and the DASS-17

Eigen-value (ordinal)	DASS-21			DASS-17		
	Eigenvalue from data	Mean of eigenvalues from parallel analysis	Upper 95th percentile of eigenvalues from parallel analysis	Eigenvalue from data	Mean of eigenvalues from parallel analysis	Upper 95th percentile of eigenvalues from parallel analysis
1	6.481885	1.749607	1.885814	5.936713	1.641377	1.773705
2	1.649158	1.605690	1.698623	1.488974	1.501426	1.593724
3	1.410058	1.500823	1.583175	1.216837	1.399425	1.473555
4	1.260653	1.413044	1.485973	1.059093	1.313195	1.378735
5	1.119023	1.335737	1.403358	.899059	1.231813	1.295945
6	.992901	1.262905	1.323083	.816808	1.161452	1.221044
7	.833116	1.196825	1.249184	.790260	1.091419	1.146133
8	.809966	1.131749	1.181814	.672332	1.030028	1.083310
9	.777705	1.071375	1.121637	.650681	.968717	1.019322
10	.700479	1.013694	1.062786	.554570	.909520	.956514
11	.638598	.958786	1.007664	.525140	.851179	.896220
12	.619486	.905441	.953616	.486496	.796644	.845344
13	.576101	.853279	.899062	.440222	.738543	.789120
14	.514772	.802496	.846184	.422979	.682736	.733027
15	.471494	.751827	.796540	.379766	.627046	.679819
16	.449180	.703096	.748118	.377949	.564222	.618043
17	.405748	.653701	.700077	.282120	.491258	.551740
18	.362971	.604337	.652196			
19	.339571	.552494	.599426			
20	.313168	.497956	.547386			
21	.273967	.435139	.491612			

*DASS* Depression Anxiety Stress Scale

As shown in Table 2, factor analysis after removal of the four items with low loadings resulted in acceptable loadings of the 17 remaining items on one factor. That one factor explained 34.9% of the variance. Similarly, parallel analysis indicated that only one factor should be retained (the right-hand side of Table 4).

### Reliability and validation tests

Reliability reflects the accuracy, precision, and consistency of a test score. Coefficient alpha is the most popular reliability coefficient. Though alpha of 0.70 can be considered reliable, the higher its value the more reliable the scale is [38]. Coefficient alpha was almost the same in the DASS-17 and the DASS-21 (0.881, and 0.883, respectively). Removal of any item from the DASS-17 would reduce its reliability (Table 5). For the DASS-17, the mean of inter-item correlations was slightly higher (0.283) than for the original scale (0.249).

**Table 5:** Means, standard deviations, internal-consistency reliability, and correlations of the DASS-21 and DASS-17 scores with stigma variables

Variables	DASS-21	DASS-17
Mean (SD)	26.7 (12.5)	21.6 (11)
Range of item-total correlations	0.304–0.661	0.400–0.695
Mean inter-item correlation	0.249	0.283
Range of α if item deleted	0.876–0.885	0.869–0.880
Coefficient α	0.883	0.881
Correlations with SSAD subscales		
Stereotype awareness	−.007	.125
Stereotype agreement	.143	.172^*^
Stereotype self-occurrence	.417^**^	.495^**^
Shame	.462^**^	.465^**^

*DASS* Depression Anxiety Stress Scale, *SSAD* Self-stigma of Alcohol Dependence Scale, *p ≤ .05, **p ≤ .000

As hypothesized, for stereotype awareness and stereotype agreement the correlations with DASS scores were all weak: from − 0.007 to 0.172 (Table 5). Also as hypothesized, for stereotype self-occurrence and for shame the correlations with DASS scores were all positive, and they were moderately strong: from 0.417 to 0.495 (Table 5). All four SSAD subscale scores correlated more strongly with DASS-17 scores than with DASS-21 scores.

## Discussion

A 17-item version of the DASS performed better than the 21-item version, and this DASS-17 provided a unidimensional index of overall psychological distress. Other studies have reported a 3-factor structure of the DASS-21 [15, 22, 23, 39–41]. However, in at least one study, items were forced to load in a manner that served the original structure of Lovibond and Lovibond (1995): for the Malay DASS-21, Nur Azma et al. (2014) developed four CFA models, although all had low fit indices. Some studies relied mainly on the criterion of an eigenvalue above 1 to decide how many factors to retain [39, 41], which can overestimate the number of factors that should be retained [36]. Further, in six studies, several items (up to 11 items) had cross-loadings i.e., loaded on more than one factor [13, 17, 25, 39–41] while some items had low loadings (below 0.3) [13, 39, 41], which can indicate model-to-data misfit i.e., a considerable number of items of the scale do not differentiate between its three main constructs.

High correlations among the DASS-21 factors have been reported in different age and ethnic groups [13, 15, 22, 28, 41]. In the study by Norton [15] those correlations were extremely high (r’s = .920–.974), which casts great doubt on the 3-factor structure of the DASS-21. Moreover, other studies indicated that the DASS-21 has a “bifactor” structure: a general factor of overall psychological distress and 3 specific factors of depression, anxiety, and stress [18, 42, 43]. The presence of a general factor resulted in a better fit and is consistent with the high correlations among depression, anxiety, and stress [13, 42, 43]. In some instances, that general factor “accounted for the greatest proportion of common variance in the DASS-21 item scores” [13, 18].

Taken together, the previous reports of a bifactor structure of the DASS-21, of high inter-factor correlations, and of item cross-loadings all support the plausibility of a 1-factor solution, which was obtained for the DASS-17 in this study. Results of parallel analysis after removal of four items with low loadings and communalities (items 2, 6, 14, and 18), indicated that the DASS-17 was unidimensional (Table 4). This is consistent with the previous finding that items 2 and 18 had very low item-discrimination indices (< 0.2) [26].

In agreement with our results, the DASS-21 was unidimensional in Latino college students near the US-Mexico border [24], nursing students in Brunei [13], and in Australian adolescents [44]. Symptoms of depression, anxiety, and anger are strongly linked and may be not easy for young people to distinguish [44]. Similarly in adults, Tran et al. (2013) found that all items of the Vietnamese DASS-21 (except item 18) loaded on one factor, and they concluded that the DASS-21 was useful only for identifying people who had both symptoms of depression and anxiety. Similar findings were reported from a study of a non-clinical population of adults in the United States [45]. The Turkish version of the DASS-21 could be used to differentiate people with depressive disorder and anxiety disorders from healthy controls, but not to differentiate patients with a diagnosis of major depression from patients with anxiety disorders [27]. Therefore, the DASS-21 can be used to assess the severity and frequency of negative emotional states, but not to measure, separately, the severity of depression or of anxiety.

Although deleting items from a scale can be associated with reduction of reliability, removal of four items in the current study decreased the value of coefficient alpha only from 0.883 to 0.881, which is considered very trivial indicating that deleted items did not really contribute to reliability of the scale. Despite the slightly lower alpha value, the DASS-17 might even perform better than the DASS-21 given the improvement of item-total correlations and inter-item correlation reported in Table 5—suggesting better convergent validity of the DASS-17. On the other side, the DASS scores correlated as hypothesized with drug-related stigma (Table 5), and stigma is strongly associated with both depression and anxiety [31]. Some drug users also have other common risk factors for both depression and anxiety: unemployment, dysfunctional relations, poor family and support networks, serious infections, financial difficulties, incarceration, and homelessness [8, 46–48]. In addition, depression, anxiety, and post-traumatic stress disorders are highly co-morbid in this group [49]. Thus it would not be at all surprising for symptoms of depression and anxiety to be strongly interwoven in this study’s participants, which might account for the unidimensionality in the present study. This argument is supported by findings of a former report, which indicated that scores of the total DASS-21 could predict depressive disorders among people with substance use disorders at sensitivity and specificity levels of 78–89% and 71–76%, respectively [19]. This could well be what has been called “common mental disorders of depression and anxiety” [5] or “anxious-depression symptomatology” [24], which were described in former studies that assessed the validity of this scale. In final, the short form of the Arabic DASS scale may not differentiate between anxiety and depression disorders (at least among people with substance use disorders) as it was intended to do; however, it might be a suitable measure of negative emotions that overlap between anxiety and depression.

## Conclusion

Even though the DASS-21 has been reported to successfully measure three different latent variables in some groups, the Arabic DASS-21 was unidimensional in the present context. Also, removing four items with poor loadings resulted in a unidimensional 17-item scale of overall psychological distress. DASS-17 scores were as reliable as the DASS-21 scores. Both the DASS-21 and DASS-17 performed as hypothesized on validation tests using the SSAD subscales. On the convergent-validation tests, correlations between the DASS-17 and stigma aspects (stereotype agreement, self-occurrence, and shame) were better than those of the DASS-21.

Because the participants were a convenience sample from a public hospital and were mainly men who used multiple drugs, they may not be representative of all Egyptian drug users. Further validation testing and studies in other populations would certainly be useful.

## Data Availability

The datasets used and/or analyzed during the current study are available from the corresponding author on reasonable request.

## Abbreviations

CFI: Comparative fit index
DASS-21: Depression Anxiety Stress Scale-21
EFA: Exploratory factor analysis
NFI: Normed fit index
RMSEA: Root mean square error of approximation
SSAD: Self-stigma of Alcohol Dependence Scale
TLI: Tucker-Lewis index

## References

[1] WHO (2017). Depression and Other Common Mental Disorders Global Health Estimates.

[2] Rotenstein LS, Ramos MA, Torre M, Segal JB, Peluso MJ, Guille C (2016). Prevalence of depression, depressive symptoms, and suicidal ideation among medical students: a systematic review and meta-analysis. JAMA..

[3] Stubbs B, Aluko Y, Myint PK, TO S (2016). Prevalence of depressive symptoms and anxiety in osteoarthritis: a systematic review and meta-analysis. Age Ageing.

[4] Falah-Hassani K, Shiri R, Dennis CL (2017). The prevalence of antenatal and postnatal co-morbid anxiety and depression: a meta-analysis. Psychol Med.

[5] Tran TD, Tran T, Fisher J. Validation of the depression anxiety stress scales (DASS) 21 as a screening instrument for depression and anxiety in a rural community-based cohort of northern Vietnamese women. BMC Psychiatry. 2013;13(24). 10.1186/1471-244X-13-24. 10.1186/1471-244X-13-24 PMC3566910 23311374

[6] Gobbi G, Atkin T, Zytynski T, Wang S, Askari S, Boruff J (2019). Association of Cannabis use in adolescence and risk of depression, anxiety, and suicidality in young adulthood: a systematic review and meta-analysisCannabis use in adolescence and risk of depression, anxiety, and suicidality in young AdulthoodCannabis use in adolescence and risk of depression, anxiety, and suicidality in young adulthood. JAMA Psychiatry.

[7] Lai HMX, Cleary M, Sitharthan T, Hunt GE (2015). Prevalence of comorbid substance use, anxiety and mood disorders in epidemiological surveys, 1990–2014: a systematic review and meta-analysis. Drug Alcohol Depend.

[8] Chen VC-H, Wu M-H, Lin T-Y, Ho Y-F, Wang H-Y, Gossop M (2015). Comparison of socio-demographic characteristics, substance, and depression among male heroin users attending therapeutic community and methadone maintenance treatment program in Nantou, Taiwan. Substance abuse treatment, prevention, and. Policy..

[9] Greenfield BL, Venner KL, Kelly JF, Slaymaker V, Bryan AD (2012). The impact of depression on abstinence self-efficacy and substance use outcomes among emerging adults in residential treatment. Psychol Addict Behav.

[10] Reid Patrice, Mann Robert, Strike Carol, Brands Bruna, Khenti Akwatu (2012). Comorbidity between psychological distress and drug use among patients in treatment centres in Jamaica: implications for policies and programme design. Texto & Contexto - Enfermagem.

[11] McKay JR (2011). Negative mood, craving, and alcohol relapse: can treatment interrupt the process?. Curr Psychiatry Rep.

[12] Witkiewitz K, Villarroel NA (2009). Dynamic association between negative affect and alcohol lapses following alcohol treatment. J Consult Clin Psychol.

[13] Teo YC, Hj Yusuf A, Alice Lim WP, Ghazali NB, Abd Rahman H, Lin N (2019). Validation of DASS-21 among nursing and midwifery students in Brunei. J Public Health.

[14] Crawford John R., Henry Julie D. (2003). The Depression Anxiety Stress Scales (DASS): Normative data and latent structure in a large non-clinical sample. British Journal of Clinical Psychology.

[15] Norton PJ (2007). Depression anxiety and stress scales (DASS-21): psychometric analysis across four racial groups. Anxiety Stress Coping.

[16] Watson D, Clark LA, Weber K, Assenheimer JS, Strauss ME, McCormick RA (1995). Testing a tripartite model: II. Exploring the symptom structure of anxiety and depression in student, adult, and patient sample. J Abnorm Psychol.

[17] Oei TPS, Sawang S, Goh YW, Mukhtar F. Using the depression anxiety stress scale 21 (DASS-21) across cultures. Int J Psychol. 2013. 10.1080/00207594.2012.755535 23425257

[18] Osman A, Wong JL, Bagge CL, Freedenthal S, Gutierrez PM, Lozano G (2012). The depression anxiety stress Scales-21 (DASS-21): further examination of dimensions, scale reliability, and correlates. J Clin Psychol.

[19] Beaufort IN, De Weert-Van Oene GH, Buwalda VAJ, de Leeuw JRJ, Goudriaan AE (2017). The depression, anxiety and stress scale (DASS-21) as a screener for depression in substance use disorder inpatients: a pilot study. Eur Addict Res.

[20] Brown TA, Chorpita BF, Korotitsch W, Barlow DH (1997). Psychometric properties of the depression anxiety stress scales (DASS) in clinical samples. Behav Res Ther.

[21] Shea TL, Tennant A, Pallant JF (2009). Rasch model analysis of the Depression, Anxiety and Stress Scales (DASS). BMC Psychiatry.

[22] Gloster AT, Rhoades HM, Novy D, Klotsche J, Senior A, Kunik M (2008). Psychometric properties of the depression anxiety and stress Scale-21 in older primary care patients. J Affect Disord.

[23] Nur Azma B, Rusli B, Quek K, Noah R (2014). Psychometric properties of the Malay version of the depression anxiety stress Scale-21 (M-DASS21) among nurses in public hospitals in the Klang Valley. Int J Collab Res Int Med Public Health.

[24] Camacho Á, Cordero ED, Perkins T (2016). Psychometric properties of the DASS-21 among Latina/o college students by the US-Mexico border. J Immigr Minor Health.

[25] Jun D, Johnston V, Kim JM, O'Leary S (2018). Cross-cultural adaptation and validation of the Depression, Anxiety and Stress Scale-21 (DASS-21) in the Korean working population. Work (Reading, Mass).

[26] Ali AM, Ahmed A, Sharaf A, Kawakami N, Abdeldayem SM, Green J (2017). The Arabic version of the depression anxiety stress Scale-21: cumulative scaling and discriminant-validation testing. Asian J Psychiatr.

[27] Yıldırım A, Boysan M, Kefeli MC. Psychometric properties of the Turkish version of the depression anxiety stress Scale-21 (DASS-21). British Journal of Guidance & Counselling. 2018:1–14.

[28] Fox RS, Lillis TA, Gerhart J, Hoerger M, Duberstein P (2018). Multiple group confirmatory factor analysis of the DASS-21 depression and anxiety scales: how do they perform in a Cancer sample?. Psychol Rep.

[29] Ali AM, Green J (2017). Differential Item Functioning of the Arabic Version of the Depression Anxiety Stress Scale-21 (DASS-21). JOJ Nurse Health Care.

[30] Swank JM, Mullen PR (2017). Evaluating evidence for conceptually related constructs using bivariate correlations. Meas Eval Couns Dev.

[31] Schomerus G, Corrigan P, Klauera T, Kuwerta P, Freybergera H, Luchta M (2011). Self-stigma in alcohol dependence: consequences for drinking-refusal self-efficacy. Drug Alcohol Depend.

[32] Wood L, Byrne R, Burke E, Enache G, Morrison AP (2017). The impact of stigma on emotional distress and recovery from psychosis: the mediatory role of internalised shame and self-esteem. Psychiatry Res.

[33] Ali AM (2018). Stigma and psychological distress among Egyptian patients with substance use disorders. Ann Ment Health.

[34] Lovibond PF, Lovibond SH. Manual for the depression anxiety stress scales (2nd ed.). Psychology Foundation, Sydney. 1995.

[35] Gignac GE (2009). Partial confirmatory factor analysis: described and illustrated on the NEO–PI–R. J Pers Assess.

[36] O’Connor BP (2000). SPSS and SAS programs for determining the number of components using parallel analysis and Velicer’s MAP test. Behav Res Methods Instrum Comput.

[37] Walker JT, Maddan S. Factor Analysis, Path Analysis, and Structural Equation Modeling. Statistics in Criminology and Criminal Justice. 3rd ed. Boston: Jones and Bartlett Publishers; 2009. p. 335.

[38] Loewenthal K, Lewis C (2001). An Introduction to Psychological Tests and Scales.

[39] Tonsing KN (2014). Psychometric properties and validation of Nepali version of the depression anxiety stress scales (DASS-21). Asian J Psychiatr.

[40] Wang K, Shi HS, Geng FL, Zou LQ, Tan SP, Wang Y (2016). Cross-cultural validation of the depression anxiety stress Scale-21 in China. Psychol Assess.

[41] SRJ M, Hall LE. The Psychometric Properties of the 21-Item Depression Anxiety Stress Scale (DASS-21) among a Sample of Young Adults. South Online J Nurs Res. 2010;10(4). https://scholar.google.com/scholar_lookup?journal=Southern+Online+J+Nurs+Res&title=the+psychometric+properties+of+21-item+depression+anxiety+and+stress+scale(dass-21)+among+a+sample+O+Young+adults&author=J+Mahmoud&author=L+Hall&volume=10&publication_year=2010&pages=1-14&.

[42] Henry JD, Crawford JR (2005). The short-form version of the depression anxiety stress scales (DASS-21): construct validity and normative data in a large non-clinical sample. Br J Clin Psychol.

[43] Vasconcelos-Raposo J, Fernandes HM, Teixeira CM (2013). Factor structure and reliability of the depression, anxiety and stress scales in a large Portuguese community sample. Span J Psychol.

[44] Patrick J, Dyck M, Bramston P (2010). Depression anxiety stress scale (DASS): is it valid for children and adolescents?. J Clin Psychol.

[45] Sinclair SJ, Siefert CJ, Slavin-Mulford JM, Stein MB, Renna M, Blais MA (2012). Psychometric evaluation and normative data for the depression, anxiety, and stress scales-21 (DASS-21) in a nonclinical sample of U.S. adults. Eval Health Prof.

[46] Link BG, Phelan JC (2001). Conceptualizing stigma. Annu Rev Sociol.

[47] Mowbray O, Scott JA (2015). The effect of drug use disorder onset, remission or persistence on an Individual’s personal social network. Am J Addict.

[48] Naji L, Dennis BB, Bawor M, Plater C, Pare G, Worster A (2016). A prospective study to investigate predictors of relapse among patients with opioid use disorder treated with methadone. Subst Abuse Treat Prev Policy.

[49] Moody L, Franck C, Bicke WK (2016). Comorbid depression, antisocial personality, and substance dependence: relationship with delay discounting. Drug Alcohol Depend.

